# Phylogenomic analysis of gastroenteritis-associated *Clostridium perfringens* in England and Wales over a 7-year period indicates distribution of clonal toxigenic strains in multiple outbreaks and extensive involvement of enterotoxin-encoding (CPE) plasmids

**DOI:** 10.1099/mgen.0.000297

**Published:** 2019-09-25

**Authors:** Raymond Kiu, Shabhonam Caim, Anais Painset, Derek Pickard, Craig Swift, Gordon Dougan, Alison E. Mather, Corinne Amar, Lindsay J. Hall

**Affiliations:** ^1^​ Gut Microbes and Health, Quadram Institute Bioscience, Norwich NR4 7UQ, UK; ^2^​ Gastrointestinal Bacteria Reference Unit, Public Health England, London NW9 5EQ, UK; ^3^​ Department of Medicine, University of Cambridge, Cambridge CB2 0QQ, UK; ^4^​ Microbes in the Food Chain, Quadram Institute Bioscience, Norwich NR4 7UQ, UK; ^5^​ Faculty of Medicine and Health Sciences, University of East Anglia, Norwich NR4 7TJ, UK

**Keywords:** *Clostridium perfringens*, food poisoning, gastroenteritis, phylogenomics, outbreaks, genomic epidemiology

## Abstract

*
Clostridium perfringens* is a major enteric pathogen known to cause gastroenteritis in human adults. Although major outbreak cases are frequently reported, only limited whole-genome sequencing (WGS) based studies have been performed to understand the genomic epidemiology and virulence gene content of outbreak-associated *C. perfringens* strains. We performed phylogenomic analysis on 109 *C*. *perfringens* isolates (human and food) obtained from disease cases in England and Wales between 2011 and 2017. Initial findings highlighted the enhanced discriminatory power of WGS in profiling outbreak *C. perfringens* strains, when compared to the current Public Health England referencing laboratory technique of fluorescent amplified fragment length polymorphism analysis. Further analysis identified that isogenic *C. perfringens* strains were associated with nine distinct care-home-associated outbreaks over the course of a 5-year interval, indicating a potential common source linked to these outbreaks or transmission over time and space. As expected, the enterotoxin *cpe* gene was encoded in all but 4 isolates (96.3 %; 105/109), with virulence plasmids encoding *cpe* (particularly pCPF5603 and pCPF4969 plasmids) extensively distributed (82.6 %; 90/109). Genes encoding accessory virulence factors, such as beta-2 toxin, were commonly detected (46.7 %; 51/109), and genes encoding phage proteins were also frequently identified. Overall, this large-scale genomic study of gastroenteritis-associated *C. perfringens* suggested that three major *cpe*-encoding (toxinotype F) genotypes underlie these outbreaks: strains carrying (1) pCPF5603 plasmid, (2) pCPF4969 plasmid and (3) chromosomal-*cpe* strains. Our findings substantially expanded our knowledge on type F *C. perfringens* involved in human-associated gastroenteritis, with further studies required to fully probe the dissemination and regional reservoirs of this enteric pathogen, which may help devise effective prevention strategies to reduce the food-poisoning disease burden in vulnerable patients, such as the elderly.

## Data Summary

1. All sequencing reads generated in this study have been deposited in the European Nucleotide Archive (ENA) (https://www.ebi.ac.uk/ena) under project PRJEB25764. Full strain information is provided in Table S1 (available in the online version of this article).

2. Reference genome *Clostridium perfringens* NCTC 8239 (genome assembly) reconstructed from sequencing reads (ENA accession number SAMEA4063013, under project PRJEB6403) and used in this study has been deposited in the ENA under assembly accession number GCA_902459515.

3. The reconstructed phylogenetic trees and associated metadata can be interactively visualized on iTOL: (a) care-home cohort (https://itol.embl.de/tree/149155215181467251528970095); (b) food-poisoning cohort (https://itol.embl.de/tree/149155215181287221528975471); (c) population structure of all 109 gastroenteritis-associated *C. perfringens* isolates (https://itol.embl.de/tree/14915521518110081531305832).

**Impact Statement**

*
Clostridium perfringens* is the second most prevalent foodborne pathogen in the UK after *Campylobacter *, causing gastroenteritis in ~80 000 cases and ~13 000 general practice consultations each year (according to the Longitudinal Study of Infectious Intestinal Disease; IID2 study). Despite the disease burden imposed by this gut pathogen, there have been only very limited studies (*n* *=*1) that have utilized whole-genome sequencing (WGS) to probe the spread and genomic characteristics of outbreak-associated strains. Here, we analysed a representative subset of 109 outbreak-associated *C. perfringens* genomes obtained in England and Wales (2011–2017) to investigate the genomic epidemiology and virulence potentials in this foodborne pathogen. We discovered that a specific clade of enterotoxin *cpe*-carrying clonal strains was associated with nine distinct outbreaks across an interval of 5 years (2013–2017) in the region of North-East England. Furthermore, we determined that ~83 % (90/109) of these isolates carried *cpe-*encoding plasmids, either pCPF5603 or pCPF4969; only 14 % of these isolates carried *cpe* on the chromosome. These data indicate that implementing WGS in *C. perfringens* surveillance laboratories would enhance outbreak tracking, and highlight the importance of further investigations exploring the dissemination and reservoirs of *C. perfringens* for effective morbidity prevention, particularly in at-risk elderly care-home residents.

## Introduction

*Clostridium perfringens* is an important pathogen known to cause disease in humans and animals [1, 2]. Notably, the pathogenesis of *C. perfringens *-associated infections is largely attributed to the wide array of toxins this species can produce, with >20 toxins currently identified [3, 4]. This Gram-positive spore former has been associated with foodborne and non-foodborne diarrhoeal diseases in humans, and preterm-necrotizing enterocolitis [5, 6].

*C. perfringens *-associated food-poisoning (FP), also termed acute watery diarrhoea, was first documented in the UK in the 1940s [7]. Typical symptoms (which occur within 8–14 h after ingestion of food contaminated with at least 10^6^ c.f.u. live bacterial cells g^−1^) include intestinal cramp and watery diarrhoea without fever or vomiting, and normally resolve in 12–24 h [8]. Importantly, *C. perfringens* is currently the second most common foodborne pathogen in the UK after *Campylobacter *, with cases often under-reported due to the frequently self-limiting nature of the illness, with current conservative estimates suggesting ~80 000 cases per annum [9–12].

In the UK, antibiotic-associated diarrhoea and non-foodborne outbreaks of *C. perfringens* diarrhoea have been frequently reported since the 1980s amongst the elderly, particularly in hospital settings [13]. With this type of illness, symptoms are more severe than for foodborne diarrhoea and are longer lasting (>3 days to several weeks), often chronic, and the infection-causing bacteria are more likely to be spread amongst people [14]. This type of *C. perfringens* infection has also been reported in elderly patients, especially those residing in care homes (CHs) in North-East England between 2012 and 2014 (83 % of the outbreaks reported from CHs) [10]. Although fatality due to *C. perfringens* diarrhoea is uncommon and the hospitalization rate is low, enterotoxigenic *C. perfringens* is estimated to cause ~55 deaths per year in England and Wales [15, 16].

The newly expanded and revised toxinotyping scheme classifies *C. perfringens* into seven toxinotypes (types A–G) according to the combination of typing toxins produced, with this classification used in this article [17]. Human cases of *C. perfringens* diarrhoea are primarily caused by type F strains (formerly classified as enterotoxigenic type A), which produce enterotoxin (CPE), encoded by the *cpe* gene [18]. This potent pore-forming toxin is reported to disrupt intestinal tight junction barriers, which is associated with intestinal disease symptoms [19]. *C. perfringens *, and associated encoded toxins, have been extensively studied with respect to disease pathogenesis, with a strong focus on animal infections [20–22]. Recent studies analysing a range of diverse *C. perfringens* strains (from both animal and human-associated infections) indicates a plastic and divergent pangenome, with a significant proportion of accessory genes predicted to be involved in virulence mechanisms and metabolism, linked to enhanced host colonization and disease initiation [3, 23]. However, studies describing human outbreak-associated *C. perfringens* infections are limited, and to date only one recent study (58 isolates) has utilized whole-genome sequencing (WGS) data to probe the genomic epidemiology of associated strains [3, 24].

We have applied in-depth genomics and phylogenetic analyses to the whole-genome sequences of 109 newly sequenced *C. perfringens* isolates associated with outbreaks or incidents of *C. perfringens* diarrhoea in England and Wales, either foodborne or non-foodborne derived. We also identified the distribution of known virulence-related determinants including toxin and antimicrobial-resistance (AMR) genes, and virulence-associated plasmid contents within food and case isolates, and probed the putative functional capabilities of the accessory genomes and virulence features within the encoded prophage genomes. Importantly, we determined that isogenic strains were associated with nine CH outbreaks in North-East England between 2013 and 2017; furthermore, we uncovered the significant involvement of location-specific virulence-plasmid-carrying *C. perfringens* in these outbreaks.

## Methods

### Bacterial isolates, genotypic identification and genomic DNA extraction

*C. perfringens* isolated from clinical cases of diarrhoea and suspected foods, when available, were referred to the reference laboratory at Public Health England (PHE), the Gastrointestinal Bacteria Reference Unit (GBRU). Identification of cultures was performed by detection of the *C. perfringens* alpha toxin and enterotoxin gene by duplex real-time PCR, as described previously [25]. Enterotoxigenic *C. perfringens *, when associated with an outbreak or incident, were then further typed for strain discrimination using fluorescent amplified fragment length polymorphism (fAFLP) analysis, as previously described [26]. In this study, 109 cultures characterized and archived by the GBRU between 2011 and 2017 were selected, representing enterotoxigenic and non-enterotoxigenic isolates from sporadic cases and outbreaks of *C. perfringens* FP and of non-foodborne *C. perfringens* diarrhoea (Table S1, available with the online version of this article). DNA was extracted from overnight *C. perfringens* cultures using a QIAsymphony DSP DNA kit (Qiagen). In brief, cultures were lysed with lysis buffer and incubated at 37 °C for 1 h before addition of 20 µl proteinase K, followed by incubation at 56 °C for 5 h until cells had visibly lysed. An additional incubation at 96 °C for 10 min followed, before addition of 0.4 mg RNAse (with incubation at 37 °C for 15 min). DNA purification was then performed on a Qiagen QIAsymphony automated platform according to the manufacturer's instructions.

### WGS, assembly and annotation

The 109 isolate cultures were subjected to a standard Illumina library preparation protocol prior to sequencing on Illumina MiSeq (PH091–PH156; *n*=41) at PHE (Colindale, London, UK) or HiSeq 2500 platforms (PH004–PH090; *n*=68) at the Wellcome Trust Sanger Institute (Hinxton, UK), with read lengths of 101 and 151 bp (paired-end reads), respectively, yielding a mean of 172-fold coverage per isolate (maximum 377-fold, minimum 41-fold; Table S1). Paired-end short-read sequences have been deposited in the European Nucleotide Archive (ENA) (https://www.ebi.ac.uk/ena) under project accession number PRJEB25764. Quality-trimmed sequencing reads were assembled using SPAdes v3.11 (PH091–PH156) to generate draft genomes with default settings, the remaining assemblies were generated at the Wellcome Trust Sanger Institute as described elsewhere (for assembly quality see Table S2) [27, 28]. Genome assemblies were annotated by Prokka v1.13 using an in-house genus-specific database that included 35 *Clostridium* species retrieved from the National Center for Biotechnology Information Reference Sequence (RefSeq) database to construct a genus-specific annotation database (Table S3) [29]. Sequences from very small contigs (contig size <200 bp) were removed prior to coding region prediction. Draft genomes were checked for sequence contamination using Kraken v1.1 (MiniKraken database) [30]. Average nucleotide identity (ANI) was assessed to validate species delineation of 109 genome assemblies (ANI >95 % compared with reference genome *C. perfringens* NCTC 8239; pyani v0.2.4) [31].

### Reconstruction of reference genome NCTC 8239

A recently PacBio-sequenced historical foodborne isolate genome, NCTC 8239, under the NCTC 3000 project was retrieved (long-read sequence accession number: SAMEA4063013) and assembled in this study with Canu v1.6 [32]. The final high-quality assembled genome consisted of 3 008 497 bp in two contigs (coverage 176-fold; contig 1 has 2 940 812 bp, contig 2 has 67 685 bp). The genome assembly of *C. perfringens* NCTC 8239 has been deposited in the ENA under accession number GCA_902459515.

### Pangenome and phylogenetic analyses

The pangenome of isolates was constructed using Roary v3.8.0 at blastp 90 % identity, adding option -s (do not split paralogs), and options -e and -n to generate core gene alignment using mafft v7.3 [33, 34]. Roary took GFF3-format annotated assemblies generated by Prokka. The pangenome includes both the core and accessory genomes; the core genome is defined as genes present in at least 99 % of the genomes, the accessory genome as genes present in <99 % of the genomes. SNP-sites v2.3.3 was used to extract SNPs from the core gene alignment for reconstructing a phylogenetic tree [35]. Phylogenetic trees were generated using FastTree v2.1.9 and annotated using iTOL v4.2 [36, 37]. FastTree was run using the generalized time-reversible (GTR) model of nucleotide evolution on 1000 bootstrap replicates to generate maximum-likelihood trees [36]. The pairwise SNP distance matrix between genomes was computed using snp-dists [38]. Bacterial population structure was analysed via the Bayesian-based clustering algorithm hierBAPS to assign lineages, implemented in R using *rhierbaps* v1.0.1 [39].

### Profiling virulence and plasmid-related sequences

The screening of toxin genes, IS elements, plasmid *tcp* loci (Table S4) and AMR genes (CARD database v2.0.0) was performed via ABRicate with 90 % identity (--minid=90) and 90 % coverage (--mincov=90) minimum cut-offs to infer identical genes [40, 41]. ariba v2.8.1 was used as a secondary approach to validate detections of both toxin and AMR genes in raw sequence fastq files [42].

### *In silico* plasmid analysis

Sequencing reads were utilized for computational plasmid prediction via PlasmidSeeker v1.0 software at default settings [43]. Plasmid prediction was based on 8514 plasmid sequences (included in the software) available in the National Center for Biotechnology Information RefSeq database (including 35 *C*. *perfringens*-derived plasmids, see Table S5). The top-ranked predicted plasmids were extracted as predicted plasmids (Table S6). Plasmid sequences from high-sequencing-coverage assemblies (>200×; single contig; *n*=12) were extracted using in-house Perl scripts, and identified by plasmid gene content including specific toxin genes and IS elements. Plasmid comparisons were performed using Easyfig v2.2.2.

### Bacterial genome-wide association study (GWAS) analysis and functional annotation

To associate subsets of genes with specific outbreaks or isolates, we used Scoary v1.6 to identify statistically related genes [44]. Cut-offs were set as ≥80 % sensitivity and 100 % specificity. Specifically, for a CH and FP subset comparison, the sensitivity cut-off was set at ≥50 % and the specificity at 100 %. Functional categories (COG categories) were assigned to genes for biological interpretation via EggNOG-mapper v0.99.3, based on the EggNOG database (bacteria) [45].

### Identification of prophages

phaster was utilized for detection of intact prophage existing in bacterial genomes (Table S7). Annotated GenBank files were submitted to the phaster web server and annotated data parsed with in-house scripts. The detection of phage was based on the scoring method and classification as described elsewhere [46]. Only intact phage regions within the genomes of completeness score >100 (of maximum 150) were analysed further, and annotated using Prokka v1.13 and Artemis for visualization in Easyfig v2.2.2 [47, 48].

## Results

### Phylogenetic analysis of gastroenteritis-associated *C. perfringens*

**Fig. 1.**
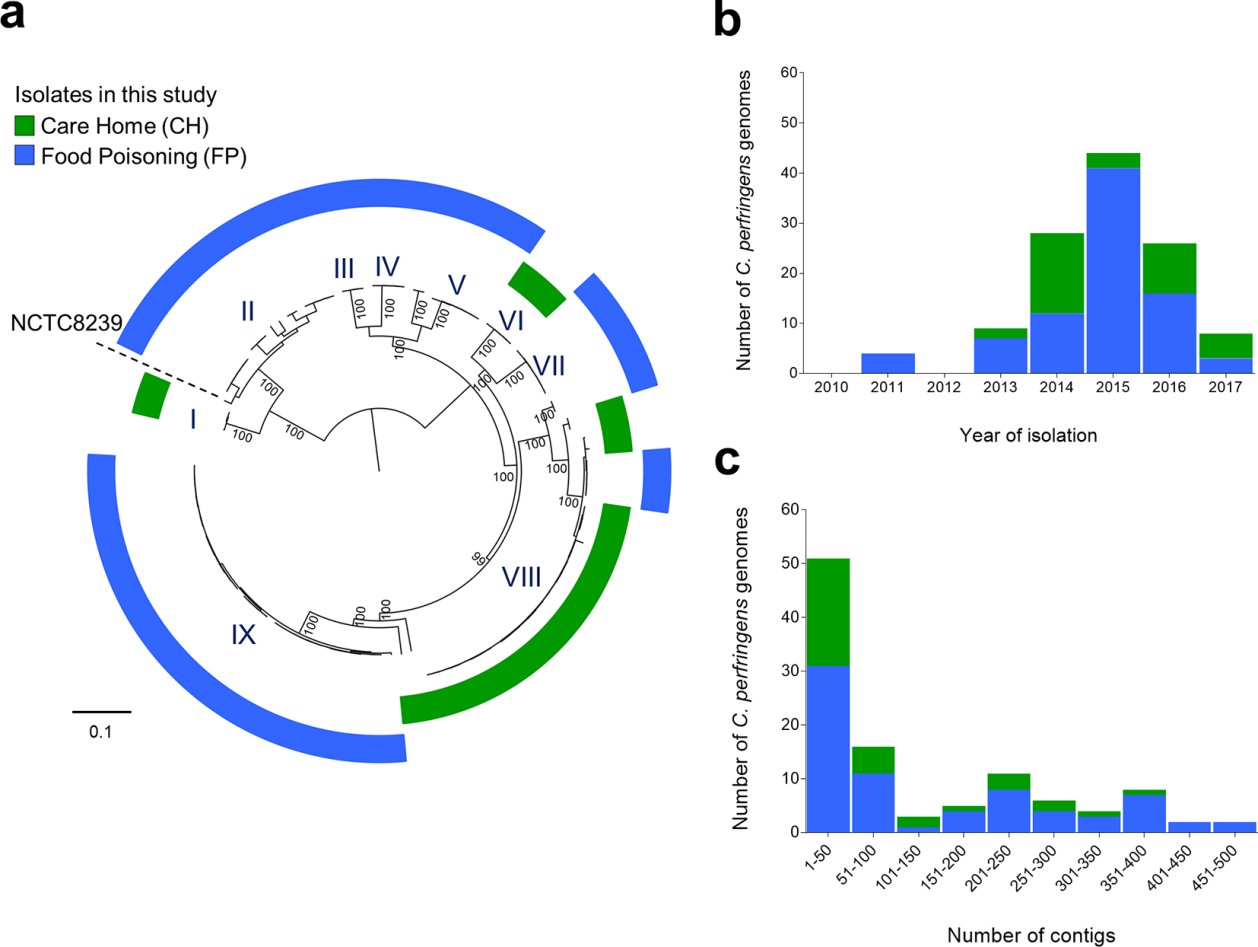
Population structure and statistics of the 109 *C*. *perfringens* genomes in this study. (a) Population structure of 109 *C*. *perfringens* isolates analysed in this study. Mid-point rooted maximum-likelihood phylogeny inferred from 73 882 SNPs identified in 110 diarrhoea-associated *C. perfringens* isolates (including NCTC 8239). The colour-coded rings indicate the cohort-specific origins of the isolates. Cluster VIII (green ring; clusters determined via hierBAPS clustering analysis) consists of primarily isolates obtained from multiple CH-associated outbreaks. Historical FP isolate NCTC 8239 was used as a reference genome as indicated in the figure. Bootstrap values are represented in the tree. Branch lengths are indicative of the estimated nucleotide substitution per site (SNPs). (b) Temporal distribution of all 109 *C*. *perfringens* genomes included in this study (2011–2017). (c) Contig count distribution of *C. perfringens* genome assemblies in this study. More than 70 % of the total assemblies are <200 contigs.

Initially, we analysed the population structure of all strains sequenced. We designated general FP isolates as FP (*n*=74), and CH-specific isolates as CH (*n*=35) (Fig. 1a, b). The quality of the genomic assemblies of draft genomes was also determined (Fig. 1c, Table S2), with >70 % of the isolate assemblies <200 contigs.

**Fig. 2.**
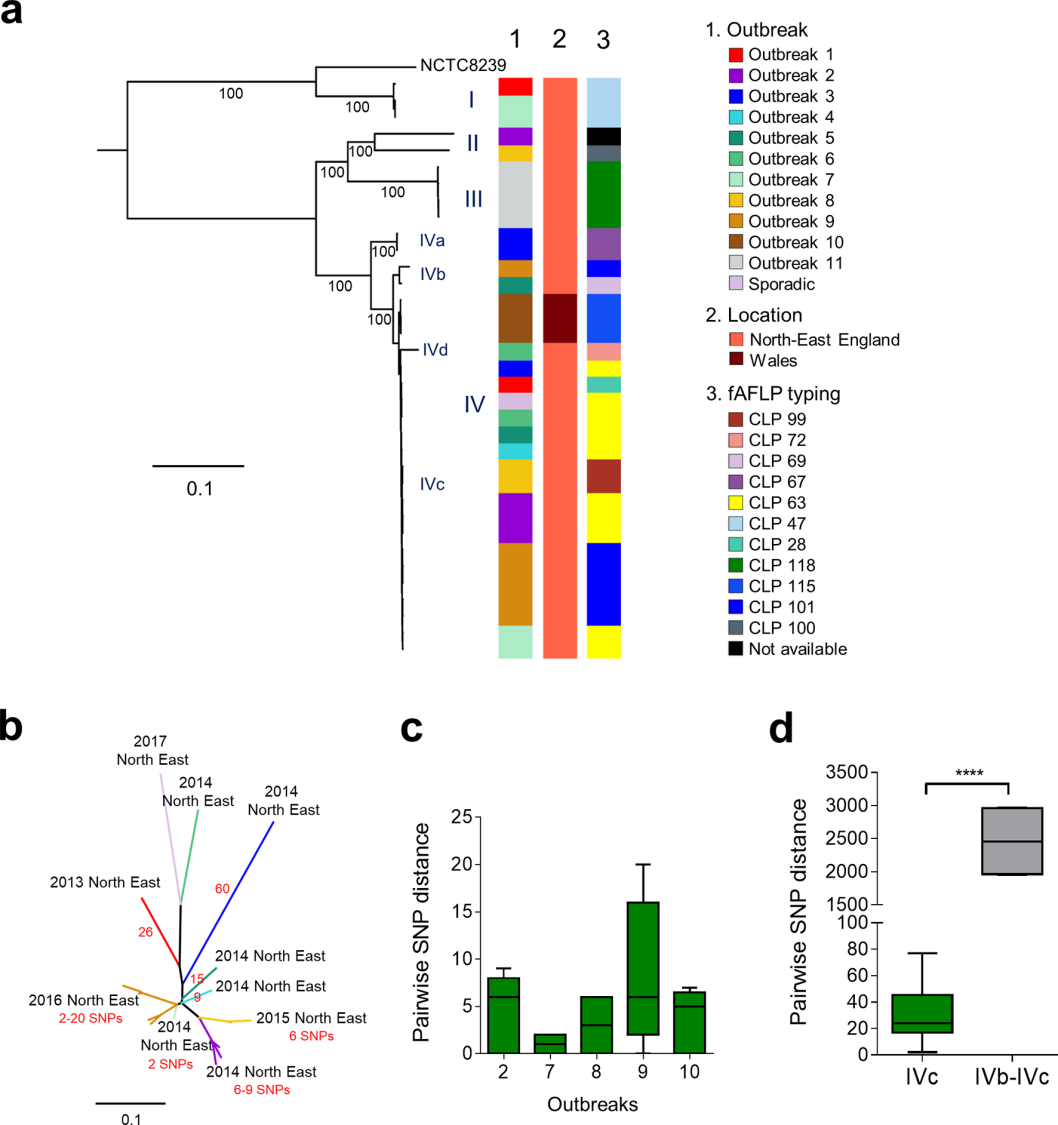
Phylogenomic analysis of CH-associated *C. perfringens* isolates. (a) Mid-point rooted maximum-likelihood phylogeny inferred from 64 560 SNPs (in core gene alignment) identified in 35 CH *C. perfringens* isolates. The colour strips indicate outbreaks (1), location of outbreaks (2) and fAFLP types (3) corresponding to the isolates. Branch lengths are indicative of the estimated SNP distance. Lineages and sub-lineages were determined via hierBAPS (level 1 and 2) clustering analysis. NCTC 8239 was used as a reference genome in this tree. Bootstrapping values are represented on the tree. (b) Unrooted maximum-likelihood tree (inferred from 191 SNPs in 18 genomes) of a sub-lineage IVc showing SNP distances between 18 North-East England derived isolates of individual outbreaks (labelled with locations, years and SNP range in the outbreaks; branches are colour-coded corresponding to individual outbreaks). SNPs between branches are indicated in red. (c) Pairwise within-outbreak core-SNP distance between isolates. (d) Pairwise outside-sub-lineage (IVb vs IVc) SNP comparison between isolates. Data were analysed using the Mann-Whitney test; ****, *P*<0.0001.

Separate analysis of CH isolates indicated four distinct phylogenetic lineages relating to CH outbreaks (Fig. 2a). Lineage I contained the reference genome NCTC 8239, a historical FP-associated *cpe*-positive isolate (originally isolated from salt beef) and three newly sequenced strains [7]. The remaining isolates clustered within three lineages (i.e. II, III and IV) that were divergent from lineage I, indicating these CH isolates might be genetically distinct from typical FP isolates as in Fig. 1a. Importantly, 18 closely related strains obtained from nine different outbreaks between 2013 and 2017, which occurred in North-East England, clustered within the same IVc sub-lineage, although not exclusively (Fig. 2a, b). SNP investigation on these IVc isolates determined mean pairwise genetic distances of 29.9±16.6 SNPs (mean±sd; range 2–77 SNPs; Fig. 2d, Table S8), suggesting an epidemiological link among these isogenic (genetically highly similar) isolates. Notably, isolates associated with specific outbreaks within sub-lineage IVc (i.e. outbreaks 2, 7, 8, 9 and 10) showed very narrow mean pairwise genetic distances of 6.6±6.6 SNPs (mean±sd; Fig. 2c), suggesting involvement of a clonal strain within these individual CH outbreaks (although a number of genetically dissimilar strains were also isolated from outbreaks 1, 2, 3, 6, 7 and 8, as shown in Fig. 2a).

This WGS analysis was also shown to have greater discriminatory power than the currently used fAFLP. The fAFLP typing (type CLP 63, displayed in yellow) failed to discriminate isolates from six different outbreaks (CH outbreaks 2–7; Fig. 2a), whilst SNP analysis clearly distinguished these strains (Fig. 2b) .

**Fig. 3.**
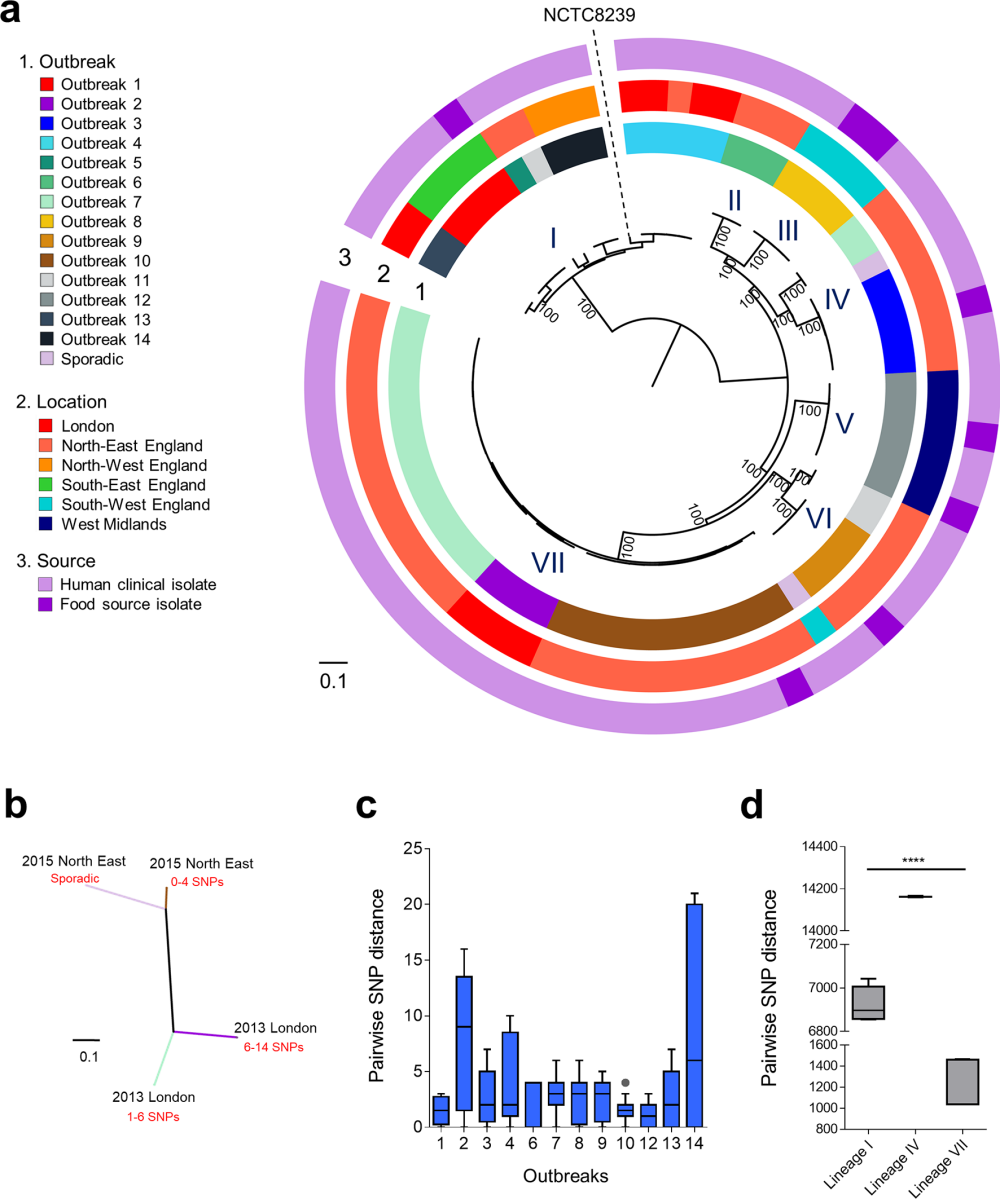
Phylogenomic analysis of FP *C. perfringens* isolates. (a) Mid-point rooted maximum-likelihood phylogeny of FP *C. perfringens* inferred from 70 613 SNPs (in core gene alignment) identified in 75 individual isolates. Lineages were determined via hierBAPS clustering analysis. Bootstrap values are represented in the tree. (b) Unrooted maximum-likelihood tree inferred from 2505 SNPs in lineage VII (31 isolates). Four distinct clusters were identified in individual outbreaks comprising genetically similar strains (labelled with locations, years and SNP range within the outbreaks; branches are colour-coded corresponding to outbreak labels). (c) Pairwise SNP distance comparison in-between isolates within outbreaks. (d) Pairwise SNP comparisons of within-major-lineage isolates in-between individual outbreaks. Lineage I, outbreaks 1,4,13 and 14; lineage IV, outbreaks 3 and 7; lineage VII, outbreaks 2, 7 and 10. Data were analysed using the Kruskal-Wallis test; ****, *P*<0.0001.

Analysis of FP isolates indicated clear separation between lineages (Fig. 3a), particularly between lineage I and the remaining lineages II–VII (pairwise mean SNP distance lineage I vs lineages II–VII, 35 165±492 SNPs; within lineage I, 5684±2498 SNPs; within lineages II–VII, 13 542±8675 SNPs). Isolates from three individual foodborne outbreaks within lineage VII appear to be highly similar (Table S9); however, further analysis indicated two different outbreaks that occurred in London (2013) were related, but somewhat distinct from isolates obtained in North-East England (2015) outbreaks (Fig. 3b).

Isolates from individual FP outbreaks (inclusive of FP lineage VII) appeared to be clonal and isogenic, as pairwise genetic distances were between 0 and 21 SNPs (mean genetic distance 2.6±2.7 SNPs; Fig. 3c), when compared to same-lineage-between-outbreaks SNP distances of >1200 SNPs (Fig. 3d). In addition, outbreak-associated food source isolates were not distinguishable from human clinical isolates (genetically similar, pairwise SNP range 0–16 SNPs) in seven individual FP outbreaks (Fig. 3a). These findings are consistent with the hypothesis that contaminated food is the main source of these *C. perfringens* FP outbreaks, which included all meat-based food stuffs, e.g. cooked sliced beef, lamb, chicken curry, cooked turkey and cooked meat (Table S1).

### Virulence gene content diversity among *C. perfringens* strains

**Fig. 4.**
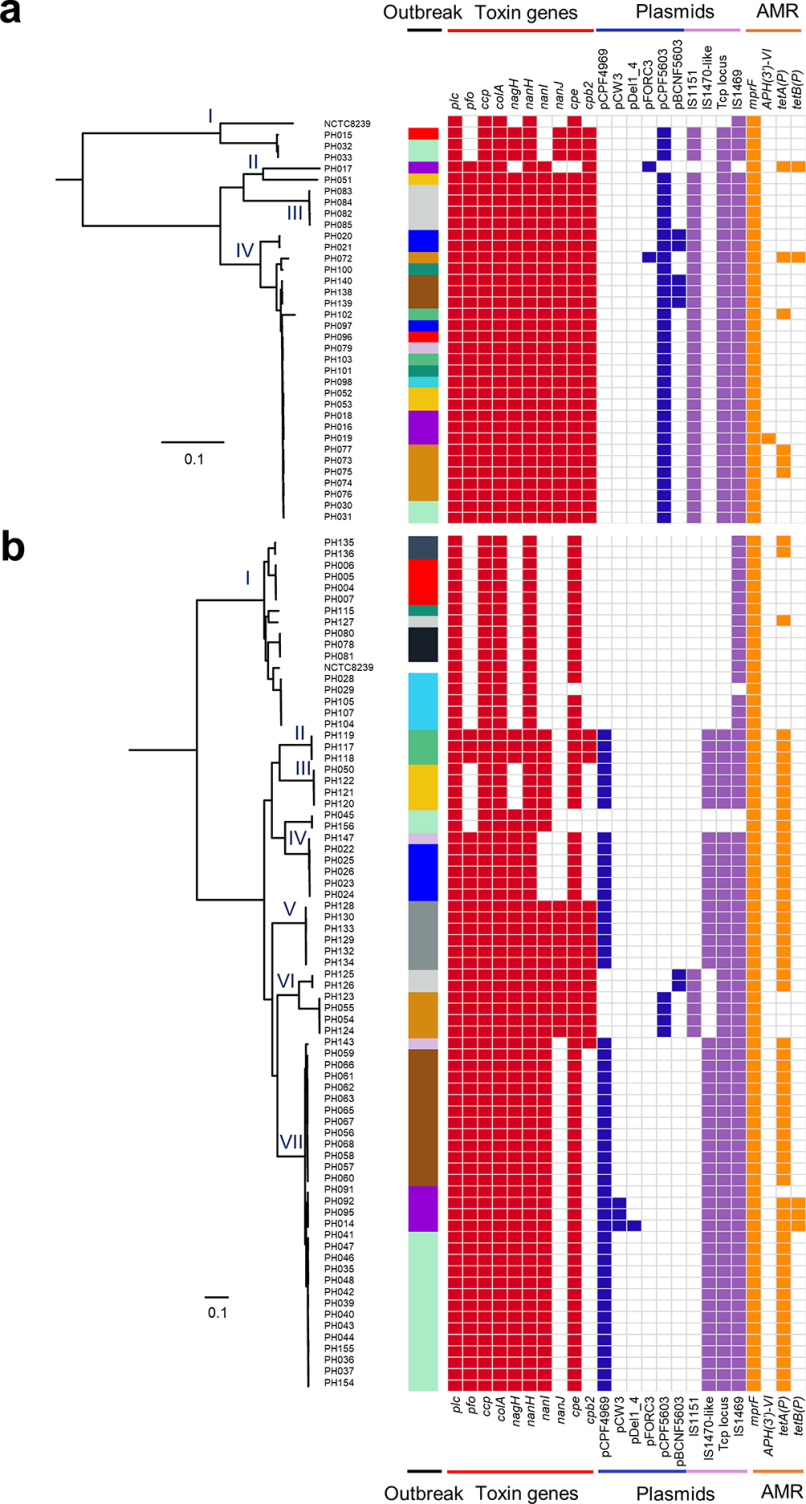
Virulence profiles of *C. perfringens* isolates including virulence plasmids. Binary heatmaps displaying the presence and absence of toxin genes, AMR genes, plasmids, plasmid-related sequences and *tcp* conjugative loci in: (a) CH isolates, (b) FP isolates. Outbreaks are colour-coded according to the colour system in previous figures. Coloured cells represent the presence of genes and white cells represent the absence of genes.

Diarrhoea symptoms associated with *C. perfringens* are primarily due to the production of the pore-forming toxin enterotoxin (CPE) by *C. perfringens* type F strains [2, 49]. Additional virulence determinants implicated in diarrhoea include sialidase (NanI), which is linked to enhanced intestinal attachment and an accessory role in enhancing CPE cell-toxicity, and also pore-forming toxin perfringolysin (PFO), a toxin known to act synergistically with alpha-toxin (a phospholipase produced by all *C. perfringens* strains) to inflict cell damage [22, 50]. Moreover, antibiotic-resistant *C. perfringens* are reported to be prevalent, particularly within poultry; thus, AMR profiles of *C. perfringens* may be linked with prolonged *C. perfringens *-associated infections, and may hamper downstream treatments strategies [51]. To probe these important virulence-associated traits, we screened both CH and FP isolates for toxin and AMR genes, based on both genome assemblies and raw sequence reads (Fig. 4).

Enterotoxin gene *cpe* was detected in all but four isolates (PH017, PH029, PH045 and PH156 were *cpe*-negative), which was confirmed by PCR, with the exception of PH029, which was initially determined to be *cpe*-positive via PCR (96.4 %; Table S1, Fig. 4).

CH isolates (mean 9.6±1.0 toxin genes per isolate) encoded significantly more toxin genes (*P*<0.001) than FP isolates (7.3±1.9 toxin genes per isolate; Fig. S1a–j). CH isolates in lineages II–IV generally possessed identical toxin profiles (Fig. 4a) including colonization-related sialidase-encoding genes *nanI*, *nanJ* and *nagH*, haemolysin PFO gene *pfo*, and *cpb2* (Fig. S1c–g)*,* which produces a vital accessory toxin beta-2 toxin (CPB2) associated with CPE-mediated pathogenesis [52]. Nevertheless, CH isolates did not harbour many acquired AMR genes, with only 6 isolates (out of 35; 17 %) encoding tetracycline-resistance genes *tet(P*), one isolate encoding aminoglycoside-resistance gene *APH(3'*) and the remaining isolates not known to carry any acquired AMR genes, other than the intrinsic AMR gene *mprF*.

FP isolates had a more variable virulence gene profile (Fig. 4b). Isolates in FP lineage I had identical toxin genes, including *cpe*, but these isolates did not encode toxins such as PFO, CPB2 and sialidase NanI, and only three isolates in this lineage carried tetracycline-resistance genes (19 %). Most isolates within FP lineages II–VII (91.4 %; 53/58) encoded *tetA(P*), with this AMR gene significantly enriched in all FP isolates (74.3 %; 55/74; *P*<0.0001; Fig. S1h), when compared to CH isolates (17.1 %; 6/35). Furthermore, most isolates in FP lineages II–VII also encoded toxin genes *cpe, nanI* and *pfo*, and 16 isolates (28 %) possessed the accessory toxin gene *cpb2*. Statistically, these FP isolates (in lineages II–VII; 8.0±1.5 toxin genes) encoded more toxin genes than those belonging to FP lineage I (4.9±0.3 toxin genes; *P*<0.0001; Fig. S1i), which may suggest increased virulence.

### Plasmid prediction and carriage investigation

The CPE toxin is responsible for the symptoms of diarrhoea in FP and non-foodborne illnesses, in the latter usually lasting >3 days and up to several weeks [2, 53]. Genetically, whilst chromosomal-encoded *cpe* strains are primarily linked to FP [54, 55], non-foodborne diarrhoea is usually associated with plasmid-borne *cpe* strains [53, 56, 57]. We performed an in-depth plasmid prediction on our datasets including a genome-wide plasmid-specific sequence search on insertion sequences IS*1151* (pCPF5603), IS*1470*-like (pCPF4969) and plasmid conjugative system *tcp* genes (Fig. 4) [58–60]. Analysis indicated that pCPF5603 plasmid carriage was associated with CH isolates (34/35 isolates; 97 %; *P*<0.0001; Fig. S1j) encoding *cpb2* and *cpe* genes; FP isolates carried predominantly pCPF4969 plasmids (45/75 isolates; 60 %) encoding *cpe* but not *cpb2* genes, whilst plasmid pCPF4969 was exclusively linked to FP lineage II–VII isolates (86.4 %; 51/59; *P*<0.0001). We observed that plasmid pCPF5603 could potentially be region specific, as 32 out of 35 CH isolates, together with the only 4 FP isolates in FP lineage VI that carried pCPF5603 plasmids, were obtained from North-East England.

**Fig. 5.**
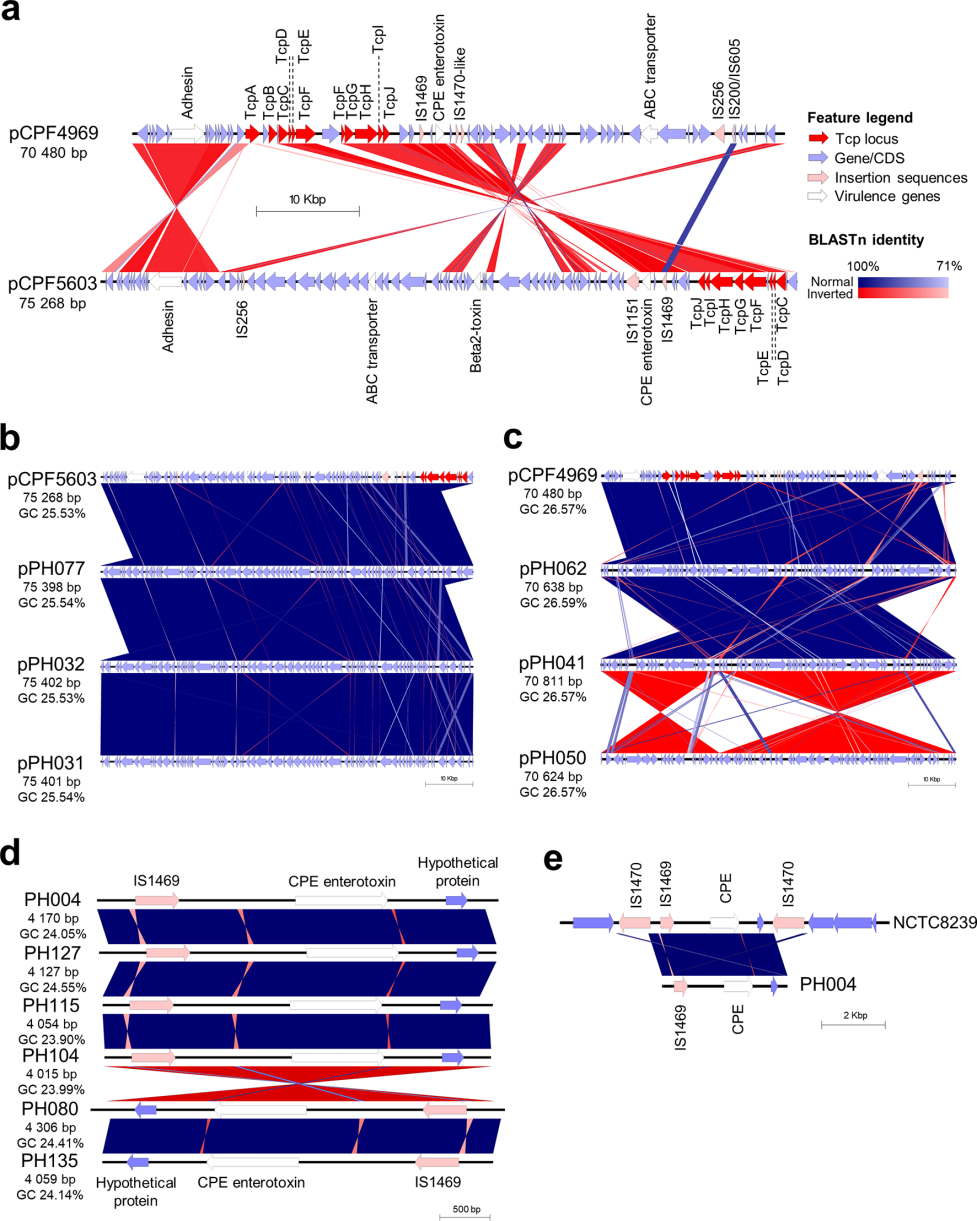
Investigations of predicted plasmids carried by CH and FP isolates. (a) Comparison of reference plasmids pCPF4969 and pCPF5603 with annotated features. CDS, Coding sequence. (b) Genomic comparison of pCPF5603 reference plasmid and predicted *cpe* plasmids from three CH isolates. (c) Plasmid comparison between pCPF4969 plasmid and FP isolate predicted *cpe* plasmids. (d) *cpe* regions (Tn*5565*) extracted computationally from FP lineage I representative isolate genomes. (e) A computationally extracted 11 kbp region of NCTC 8239 that encodes Tn*5565* (including *cpe* and flanking IS*1470* elements) compared to the predicted *cpe* region from PH004.

To further examine the predicted plasmids, we extracted plasmid sequences from genome assemblies of three isolates per CH or FP group, and compared them with reference plasmids (Fig. 5a–c). The extracted plasmid sequences closely resembled the respective reference plasmids, with near-identical nucleotide identity (>99.0 %), plasmid size and G+C content (Fig. 5b–c); thus, supporting the findings that these two intact plasmids (pCPF4969 and pCPF5603) are present in these isolates.

Although chromosomal-*cpe* strains are considered as the primary strain type to be associated with FP, our dataset demonstrated that plasmid-*cpe C. perfringens* strains were predominantly associated with FP (82.6 %; 90/109), with only 17.4 % (19/109) FP isolates encoding a copy of *cpe* with no plasmid detected. Putatively, plasmid transfer may have occurred in CH outbreak 7 isolates (*n*=4), as two isolates resided within lineage IV (PH030 and PH031), whilst the other two isolates nest within the genetically distant CH lineage I (PH032 and PH033; genetic distance >10 000 SNPs); however, all four isolates harboured the conjugative *cpe*-encoding plasmid pCPF5603 (Fig. 4a). CH outbreaks 1 and 8 also had dissimilar strains (nested within separate lineages) with identical pCPF5603 plasmids. This analysis indicates that multiple distinct strains, but carrying the same *cpe* plasmid, may be implicated in these CH outbreaks, which may potentially be attributed to plasmid transfer among *C. perfringens* strains via conjugation (*cpe*-positive to *cpe*-negative strains) [61].

### A distinct phylogenetic lineage of chromosomal-*cpe C. perfringens* strains

Previous studies have demonstrated that *C. perfringens* with chromosomally encoded *cpe* are genetically divergent from plasmid-*cpe* carriers. Within the FP phylogeny there was a distinct lineage of isolates (FP lineage I; *n*=17) that appear to encode *cpe* chromosomally. These isolates had significantly smaller genomes (genome size 2.95±0.03 Mb vs 3.39±0.08 Mb outside-lineage; *n*=93; *P*<0.0001; Table S10), were similar to historical chromosomal-*cpe* isolate NCTC 8239 in the same lineage I (ANI ≥99.40 %) and appeared to lack plasmids [57, 59]. To further investigate this hypothesis, the *cpe*-encoding region was extracted from genome assemblies of representative isolates in lineage I (*n*=6), and comparative genomics was performed (Fig. 5d). These consistently smaller (~4.0–4.3 kb) contigs were almost identical in nucleotide identity (>99.9 %) when compared with the *cpe*-encoding region of chromosomal-*cpe* strain NCTC 8239, confirming that these isolates possessed the same *cpe* genomic architecture as transposable element Tn*5565* in NCTC 8239 (Fig. 5e). We observed that PH029 was the only outbreak isolate not detected to encode *cpe* within the lineage I outbreak cluster, despite having a clonal relationship with PH028, PH104, PH105 and PH107 (FP outbreak 4). This suggests Tn*5565* loss may have occurred due to extensive sub-culturing (this is supported by initial PCR results being *cpe*-positive; see Table S1). Analysis also indicated that *cpe* was closely associated with IS*1469* independent of where it was encoded, as this insertion sequence was detected exclusively in all *cpe*-encoding genomes (100 %; Fig. 4a, b).

### Virulence potentials in the accessory genome

The 110 isolate *C. perfringens* pangenome consisted of 6219 genes (including NCTC 8239; Fig. S2), 1965 core genes (31.6 %) and 4254 accessory genes (68.4 %); with ~30–40 % genes in any individual strain encoding accessory genes, potentially driving evolution and genome restructuring. Mobile genetic elements including plasmids, genomic islands and prophages could potentially contribute to virulence, given the plasticity of the genome. To explore this in more detail, we further analysed the accessory genome, comparing different subsets of *C. perfringens* isolates. We first identified subset-specific genes using a bacterial genome-wide association study (GWAS) approach, with these genes further annotated and categorized under COG classes into three comparison groups: (1) CH versus FP; (2) FP outbreaks; (3) FP lineage I, FP lineage II–VII and CH-FP plasmid-CPE isolates (Fig. S3a–d).

Phosphotransferase system (PTS)-related genes (*n*=4) were encoded exclusively in CH isolates (present in 26/35 CH isolates; Fig. S3a, Table S11). These genes may contribute to the isolates’ fitness to utilize complex carbohydrates (COG category G) in competitive niches, like the gastrointestinal tract [62]. Heat-shock protein (Hsp70) DnaK co-chaperone was annotated in the FP-specific accessory genome (present in 57/74 FP isolates), which may be involved in capsule and pili formation that may facilitate host colonization [63–65].

Accessory genes specific to FP outbreaks were variable (Fig. S3b, c, Table S12), but three annotated functional classes were conserved, L (replication, recombination and repair), M (cell wall/membrane/envelope biogenesis) and V (defence mechanisms). Prominent genes detected in all isolates included phage-related proteins (*n*=49) (L, M and S), glycosyltransferases (*n*=37) (M), restriction modification systems (*n*=16) (V), transposases (*n*=9) (L) and integrases (*n*=8) (L).

Correspondingly, less group-specific accessory genes of FP lineage I isolates were compared with isolates in lineages II–VII (Fig. S3c, Table S13). Notably, multidrug transporter ‘small multidrug resistance’ genes were exclusively detected in FP lineage I isolates, whereas ABC transporters were more commonly encoded in plasmid-carrying isolates. The Mate efflux family protein gene was detected exclusively in lineage II–VII isolates.

### Prophage detection in *C. perfringens*

Phages are important drivers of bacterial evolution and adaptation, and the presence of prophage within bacterial genomes is often associated with enhanced survival and virulence, e.g. sporulation capacity and toxin secretion [66–68]. We identified a total of 7 prophages in all 109 *C*. *perfringens* isolates (Fig. S4a, b). Further exploration of the virulence and survival-enhancing genes (Fig. S4c) encoded in these predicted prophage-encoding regions revealed the presence of virulence-related enzyme sialidase NanH (promotes colonization), putative enterotoxin EntB, various ABC transporters (linked to multidrug resistance) and toxin-linked phage lysis holin (with a probable link to toxin secretion) [69–72]. No differences in the number of prophages carried were detected between CH and FP isolates (Fig. S4d, e).

## Discussion

*C. perfringens* is often associated with self-limiting or longer-term gastroenteritis; however, our knowledge of the genomic components that may link to disease symptoms or epidemiological comparisons between outbreaks are limited. In this study, WGS data and in-depth genomic analysis on a representative subset of 109 gastrointestinal outbreak-associated *C. perfringens* isolates revealed potential phylogenetic clusters linked to plasmid carriage, and specific virulence determinants, which were strongly associated with outbreak isolates.

In the context of disease control, it is important to gain detailed genomic information to predict transmission modes for pathogens. Our analysis of CH isolates indicated a specific persistent clone may have been responsible for up to nine individual gastrointestinal outbreaks in North-East England over the 2013–2017 period. A previous study indicated the presence of persistent identical *C. perfringens* genotypes within CH settings, with several individuals harbouring identical strains throughout a 9-month sampling period; however, none of these isolates were positive for the *cpe* gene [73]. Furthermore, although CH isolates were defined as ‘non-foodborne’ according to local epidemiological investigations, as no food samples were identified as *C. perfringens *-positive, these outbreaks may have resulted from contaminated food products not sampled. Indeed, a recent investigation into fresh meat products (>200 samples) demonstrated significant contamination (beef, ~30 %; poultry and pork, both ~26 %), with 90 % of strains *cpe-*positive, suggesting food chain(s) or farmed animals as potential reservoirs of enterotoxigenic *C. perfringens* [57, 74]. Furthermore, 18 % prevalence of *cpe-*positive *C. perfringens* strains had previously been reported in food handlers’ faeces (confirmed via PCR), denoting a potential role of the human reservoir.

A total of 4 out of 109 outbreak-associated strains were *cpe-*negative, suggesting secondary virulence genes (e.g. *pfo* and *cpb2*) may be associated with *C. perfringens *-associated gastroenteritis. A recent WGS-based study on *C. perfringens* FP outbreaks in France determined that ~30 % of isolates were *cpe-*negative (13/42) [24], indicating this gene may not be the sole virulence determinant linked to *C. perfringens* gastroenteritis. Although we observed fewer *cpe-*negative strains, this may be due to our targeted *cpe-*positive isolation strategy (standard practice at PHE). Thus, to determine the importance and diversity of *cpe-*negative strains in FP outbreaks will require untargeted isolation schemes in the future.

Typical *C. perfringens *-associated FP was previously thought to be primarily caused by chromosomal-*cpe* strains. This is linked to their phenotypic capacity to withstand high temperatures (via production of a protective small acid soluble protein) and high salt concentrations during the cooking process, in addition to the shorter generation time, when compared to plasmid-*cpe* carrying strains [55, 75]. Previous studies have indicated that these strains commonly assemble into distinct clusters that lack the *pfo* gene, which we also noted in the FP lineage I data from this study [24, 76, 77]. Plasmid-borne *cpe-*carrying strains (pCPF4969 or pCPF5603) have also been associated with previous FP outbreaks, with a previous study indicating that pCPF5603-carrying strains were associated with FP in Japanese nursing homes (7/9 isolates) [58]. However, plasmid-*cpe* outbreaks appear to be a relatively uncommon occurrence; thus, it is surprising that most outbreak-isolated strains in this study (81.7 %; 89/109) carried a *cpe* plasmid [49, 54]. Notably, plasmid-*cpe* strains can cause diverse symptoms, including short-lived FP and long-lasting non-foodborne diarrhoea, which suggests a role for additional factors in the disease pathogenesis. The gut microbiome is expected to be an important host factor, and previous studies have reported that CH residents have a less diverse and robust microbiota when compared to those residing in their own homes (including individuals colonized with *C. perfringens *), which may correlate with impaired ‘colonization resistance’ [51, 73, 78].

Chromosomal *cpe* is reported to be encoded on a transposon-like element, Tn*5565* (6.3 kb, with flanking copies of IS*1470*), which can form an independent and stable circular form in culture extracts (losing both copies of IS*1470*) [59, 61]. This transposition element TN*5565* was commonly thought to be integrated into the chromosome at a specific site as a unit in chromosomal-*cpe* strains. Our computational analysis failed to detect any *cpe,* IS*1469* (*cpe-*specific) and IS*1470* (Tn*5565*-specific) in the high-sequencing-coverage PH029 genome (317× sequencing depth/coverage), indicating Tn*5565* can be lost, which is supported by an inability to detect *cpe* in the raw sequencing reads. However, IS*1470* may not have been correctly assembled during the genome assembly process due to the repetitive nature of those sequences (a known caveat of short-read sequencing).

As WGS provides enhanced resolution to identify outbreak-specific clonal strains, our study highlights the importance of implementing WGS for *C. perfringens* profiling in reference laboratories, in place of the conventional fAFLP [76, 79, 80]. Routine *C. perfringens* surveillance of the CH environment and staff could prove critical for vulnerable populations, as outbreaks could rapidly spread, and this approach could potentially pinpoint the sources of contamination and eventually eliminate persistent *cpe* strains in the environment [73].

Our data highlight the genotypic and epidemiology relatedness of 109 *C*. *perfringens* strains isolated from FP cases from across England and Wales, and indicate the potential circulation of disease-associated strains, and the impact of plasmid-associated-*cpe* dissemination, linked to outbreak cases. This study suggests that further WGS phylogenetic and surveillance studies of diversely sourced *C. perfringens* isolates are required for us to expand our knowledge of clonal dissemination and potential regional reservoirs of FP-associated strains, so that efficient intervention or prevention measures can be devised, particularly in vulnerable communities, including older adults residing in CHs.

## Data bibliography

1. Kiu R. European Nucleotide Archive, accession no. PRJEB25764 (2019).

2. NCTC 3000 project, European Nucleotide Archive, accession no. SAMEA4063013 (2018).

3. Kiu R. European Nucleotide Archive, accession no. GCA_902459515 (2019).

4. Jia B, Raphenya AR, Alcock B, Waglechner N, Guo P, Tsang KK, Lago BA, Dave BM, Pereira S, Sharma AN, *et al*. CARD: Comprehensive Antibiotic Resistance database, v2.0.0 February 2018 release (2018).

5. The accession numbers of 35 *Clostridium* genomes used for genome annotation in this study are provided in Table S3.

6. The accession numbers of *C. perfringens*-associated toxin genes, plasmid-specific conjugative loci and insertion sequences used in this study are provided in Table S4.

## Supplementary Data

Supplementary File 1

Supplementary File 2
