# Supplementary material for: Phylogenomic analysis of gastroenteritis-associated Clostridium perfringens in England and Wales over a 7-year period indicates distribution of clonal toxigenic strains in multiple outbreaks and extensive involvement of enterotoxin-encoding (CPE) plasmids
Source: Microb Genom. 2019 Sep 25;5(10):e000297. doi: 10.1099/mgen.0.000297 (PMC6861862; doi:10.1099/mgen.0.000297)
Supplement: Supplementary File 2 [file mgen-5-297-s002.pdf]

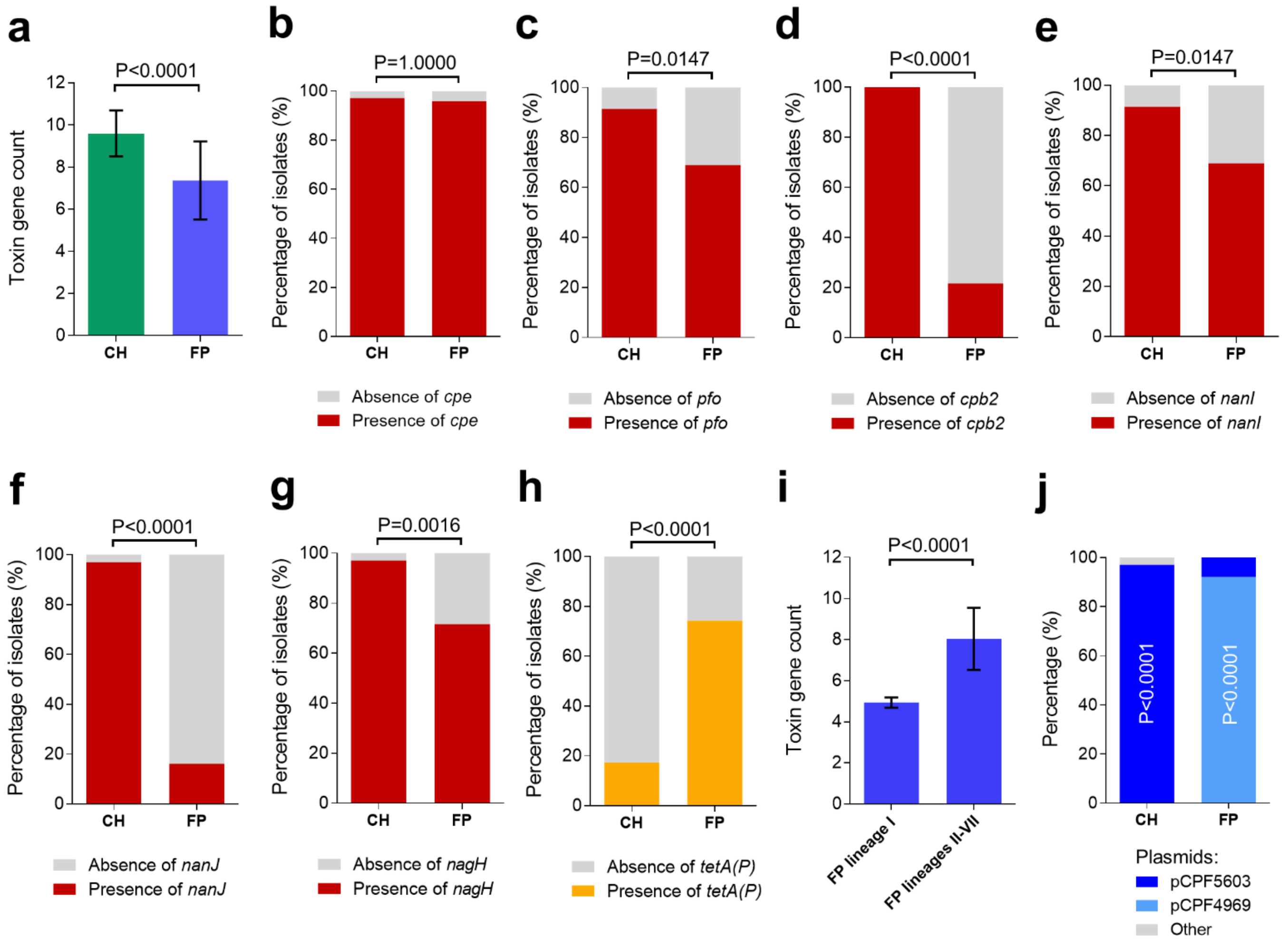

Fig. S1. Comparisons of toxin gene count, enrichments of toxin, AMR gene and plasmids
(a) Toxin gene count comparison between CH and FP isolates. Data: mean ± S.D. Data were analysed using the Mann-Whitney test. Enrichments of variable toxin genes (B-G): **(b)** *cpe* **(c)** *pfo* **(d)** *cpb2* **(e)** *nanI* **(f)** *nanJ* **(g)** *nagH* **(h)** Percentage of AMR gene *tetA(P)*-carrying isolates in CH and FP groups. **(i)** Toxin gene count comparison of isolates in FP lineage I and lineages II-VII. Data: mean ± S.D. Data were analysed using the Mann-Whitney test. **(j)** Main virulence plasmids found in CH and FP isolates. Data: percentage of isolates that carry either pCPF5603 or pCPF4969 plasmids (excluding isolates not detected to carry any plasmids). Data from (B)-(H) and (J) were analysed using Fisher's exact test (two-tailed).

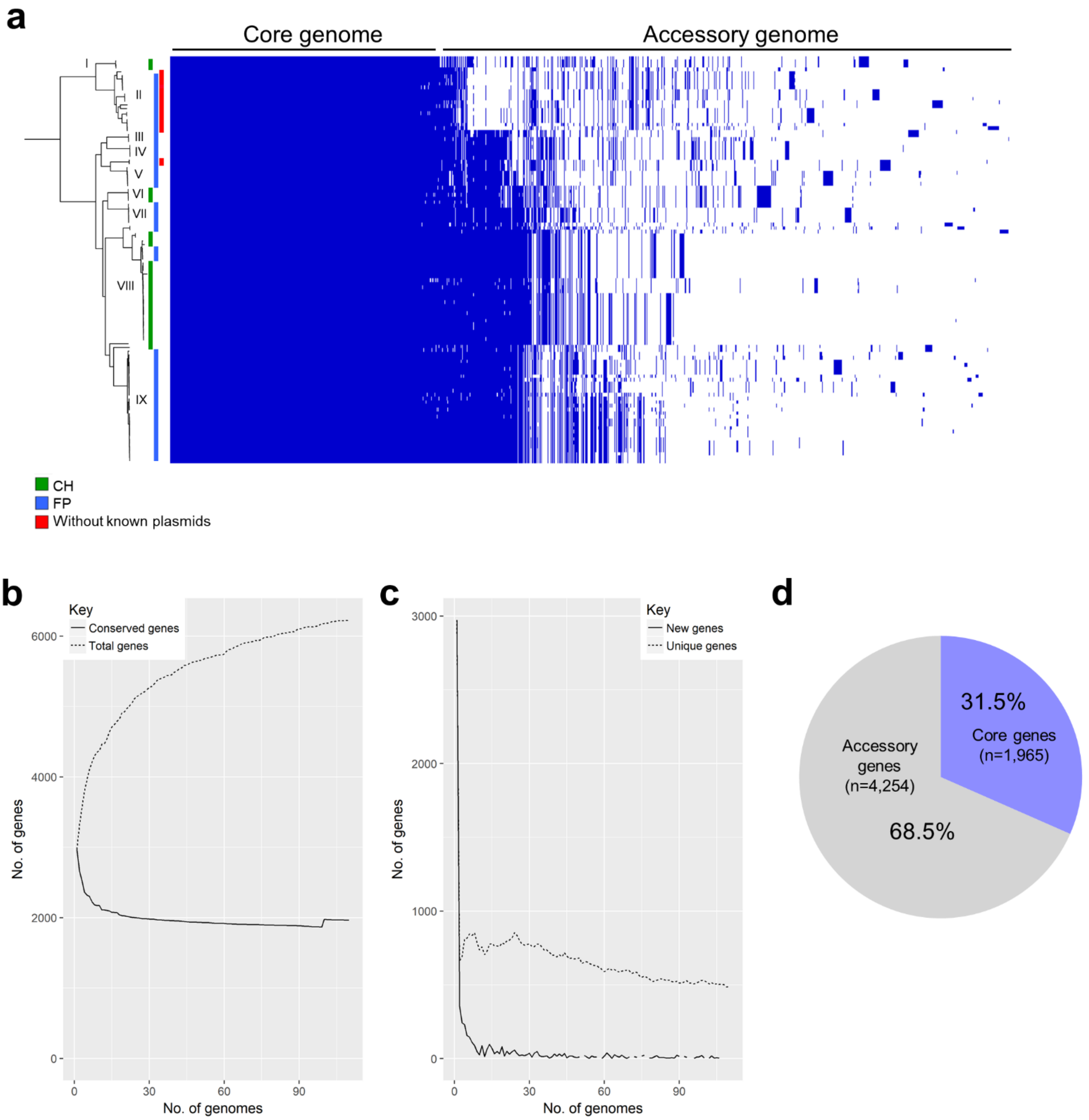

Fig. S2. Pangenome analysis of 110 *C. perfringens* isolates in this study
(a) Linearised pangenome of 110 *C. perfringens* strains in this study including reference genome NCTC 8239. Visualised by Phandango. Statistics showing addition of **(b)** conserved genes vs total genes and **(c)** New genes vs unique genes per genome during pangenome construction. **(d)** Proportion of core and accessory genes in the pangenome.

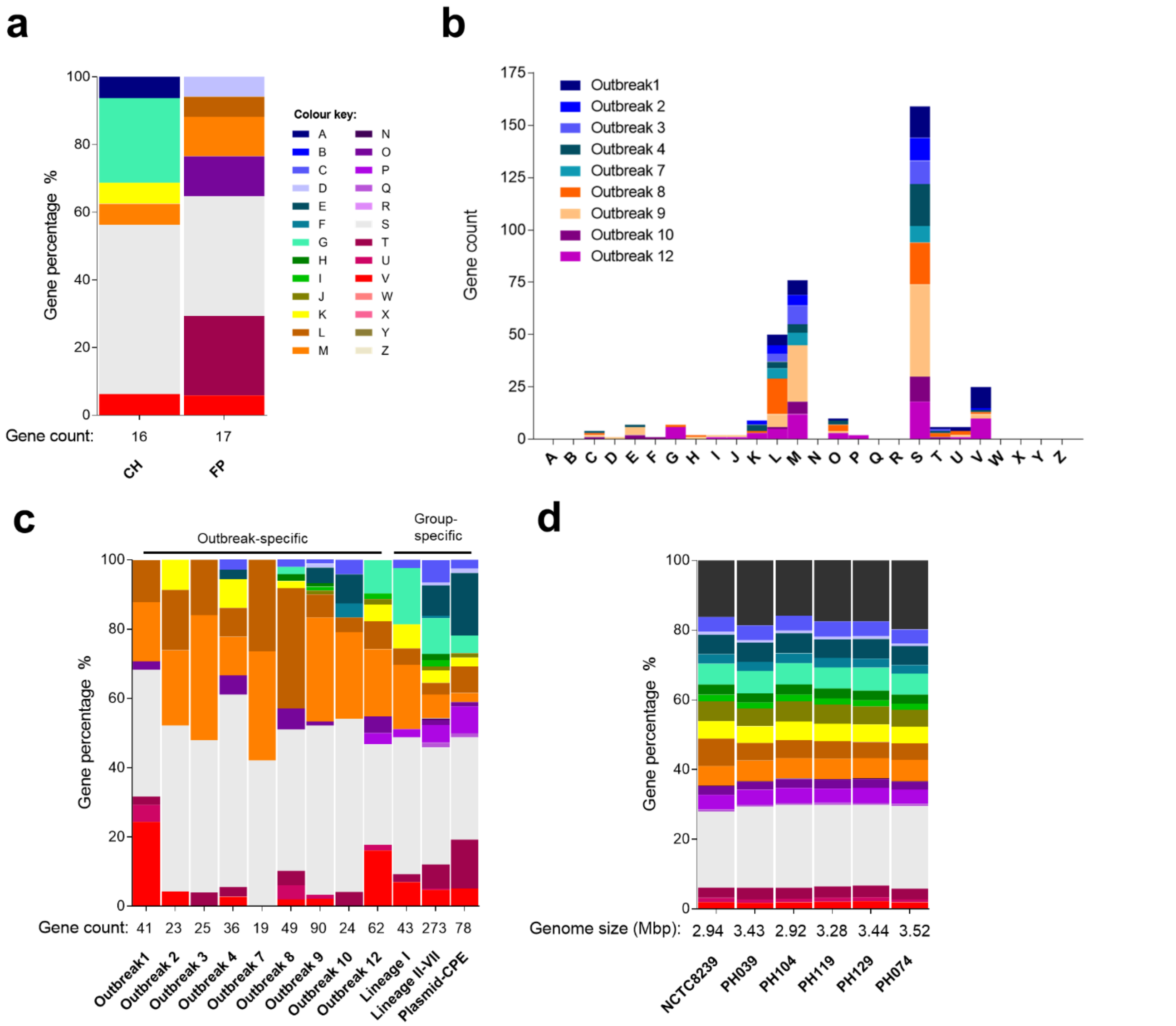

Clusters of Orthologous Groups (COG)

A: RNA processing & modification
B: Chromatin structure & dynamics
C: Energy production & conversion
D: Cell cycle control, cell division, chromosome partitioning
E: Amino acid transport & metabolism
F: Nucleotide transport & metabolism
G: Carbohydrate transport & metabolism
H: Coenzyme transport & metabolism
I: Lipid transport & metabolism
J: Translation, ribosomal structure & biogenesis
K: Transcription
L: Replication, recombination & repair
M: Cell wall/membrane/envelope biogenesis

N: Cell motility
O: Post-translational modification, protein turnover, & chaperones
P: Inorganic ion transport & metabolism
Q: Secondary metabolites biosynthesis, transport, & catabolism
R: General function prediction only
S: Function unknown
T: Signal transduction mechanisms
U: Intracellular trafficking, secretion, and vesicular transport
V: Defense mechanisms
W: Extracellular structures
X: Mobilome, phages and transposons
Y: Nuclear structure
Z: Cytoskeleton

Fig. S3. Functional analysis of *C. perfringens* genomes
(a) Subset-specific accessory genes comparison CH vs FP isolates. **(b)** Outbreak-specific isolate gene counts of COG-assigned food-poisoning accessory genomes. **(c)** Functional classification (COG) of representative outbreak- and group-specific accessory genes in FP isolates. **(d)** Whole-genome functional analysis of representative *C. perfringens* isolates including reference genome NCTC 8239. Box (bottom): specific functional description of COG assignments used in this study.

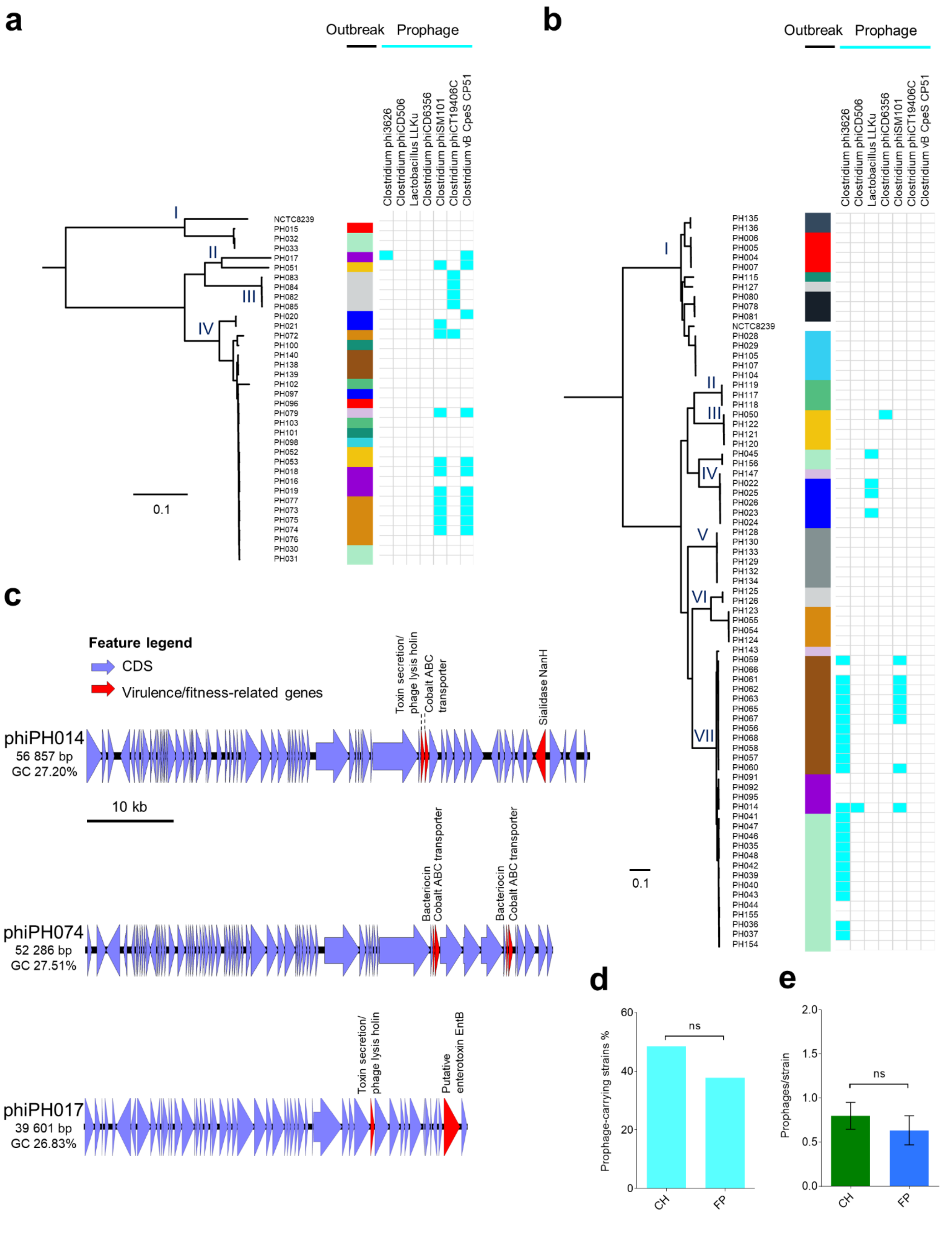

Fig. S4. Predicted prophage genomes in gastroenteritis-associated *C. perfringens* isolates
(a) Heatmap showing distribution of predicted prophage genomes in care-home associated *C. perfringens* isolates aligned with phylogenetic tree. **(b)** In food-poisoning associated *C. perfringens* isolates. Cyan-coloured cells indicate presence of predicted prophages while white indicates absence. **(c)** Genome features including fitness-related genes predicted in representative prophage genomes. **(d)** Proportion of prophage-carrying strains in both groups CH and FP. Data: ns=non-significant **(e)** Statistics on number of prophage per *C. perfringens* strains. Data: mean ± S.E.M.; ns=non-significant.
